# The Clinicopathologic and Prognostic Significance of Programmed Cell Death Ligand 1 (PD-L1) Expression in Patients With Prostate Cancer: A Systematic Review and Meta-Analysis

**DOI:** 10.3389/fphar.2018.01494

**Published:** 2019-01-24

**Authors:** Yan Li, Qingying Huang, Yaoyao Zhou, Meizhi He, Jianhong Chen, Yubo Gao, Xue Wang

**Affiliations:** ^1^Department of Urology, Zhujiang Hospital, Southern Medical University, Guangzhou, China; ^2^The Second School of Clinical Medicine, Southern Medical University, Guangzhou, China; ^3^Department of Plastic and Cosmetic Surgery, Nanfang Hospital, Southern Medical University, Guangzhou, China

**Keywords:** prostate cancer, PD-1/PD-L1, prognostic, clinicopathologic, meta-analysis

## Abstract

**Background:** Programmed cell death ligand 1 (PD-L1) expression has been shown to correlate with poor prognosis in diverse human cancers. However, limited data exist on the prognostic and clinicopathologic significance of PD-L1 expression in prostate cancers (PCa), and the curative effect of anti-PD-1/PD-L1 therapy remains controversial. In this systematic review and meta-analysis, we aimed to evaluate the prognostic and clinicopathologic value of PD-L1 in PCa.

**Methods:** We performed a systematic literature search in the PubMed, Cochrane Library, EMBASE, Web of Science, and SCOPUS databases up to July 21st, 2018. Pooled prevalence of PD-L1 in PCa was calculated using Freeman-Tukey double arcsine transformation by R software version 3.5.0. The data from the studies were examined by a meta-analysis using Review Manager software 5.3 to calculate pooled hazard ratios (HRs) and pooled odds ratios (ORs) with 95% confidence intervals (CIs) to estimate the prognostic and clinicopathologic value of PD-L1 in PCa. Heterogeneity was tested by the Chi-squared test and *I*^2^ statistic.

**Results:** Five studies with 2,272 patients were included in this meta-analysis. The pooled prevalence of PD-L1 in PCa was 35% (95% CI 0.32 to 0.37). Both PD-L1 expression (*HR* = 1.78; 95% CI 1.39 to 2.27; *p* < 0.00001) and PD-L1 DNA methylation (*HR* = 2.23; 95% CI 1.51 to 3.29; *p* < 0.0001) were significantly associated with poor biochemical recurrence-free survival (BCR-FS). PD-L1 tended to have high expression levels in high Gleason score cases (*OR* = 1.54; 95% CI, 1.17 to 2.03; *P* = 0.002) and androgen receptor-positive cases (*OR* = 2.42, 95% CI 1.31 to 4.50; *P* = 0.005). However, PD-L1 had relatively weak correlation with age, pathologic stage, lymph node metastasis and preoperative PSA level.

**Conclusions:** This meta-analysis confirms the negative prognostic significance of PD-L1 expression and mPD-L1 in PCa patients. Additionally, PD-L1 has a statistically significant correlation with Gleason score and androgen receptor status, while the correlations with age, pathologic stage, lymph node metastasis, and preoperative PSA level were not statistically significant. However, the number of included studies is too small to make the conclusions more convincing, so more retrospective large-cohort studies are expected for the further confirmation of these findings.

## Introduction

As a malignancy in the male reproductive system, prostate cancer (PCa) was not only the second most common cancer in males worldwide both in 2012 (1,112,000 new cases; 15.0%) (Ferlay et al., 2015) and 2018 (1,276,100 new cases; 13.5%) (Ferlay et al., 2018), but also the most common cancers among males in the United States (164,690 new cases; 19%) in 2018 (Siegel et al., 2018). Overall, PCa was the fifth leading cause of cancer-related death in men worldwide (307,000 deaths; 6.6%) in 2012 (Ferlay et al., 2015), while it became the fourth leading cause of cancer-related death in men worldwide (359,000 deaths; 6.7%) in 2018 (Ferlay et al., 2018). Furthermore, PCa was the second leading cause of cancer-related death in men in the United States (29,430 deaths; 9%) in 2018 (Siegel et al., 2018). PCa incidence rates increased, whereas PCa mortality rates declined in most countries in recent years, especially in more developed nations (Wong et al., 2016). Due to earlier detection by prostate-specific antigen (PSA) testing and advances in treatment, the mortality of PCa rapidly declined by 52% from 1993 to 2015 (Siegel et al., 2018). For all cancers combined, 5-year relative survival rates is highest for prostate cancer patients with localized disease (99%) during the recent time period (2007–2013) (Siegel et al., 2018), but declines to 28% for those at distant stage (Miller et al., 2016). Clinical decisions vary in the extent of disease, risk of recurrence and patient characteristics, so active surveillance is recommended for less aggressive tumors as well as older patients and/or those with severe comorbidities. Treatment options for early-stage localized prostate cancer include radical prostatectomy, external beam radiotherapy, androgen deprivation therapy (ADT), chemotherapy, bone-directed therapy, radiation, while a combination of the above therapies is used for advanced disease (Horwich et al., 2010; Miller et al., 2016). Current therapies in metastatic castration-resistant prostate cancer (mCRPC) include androgen receptor (AR)-targeted therapy, chemotherapy, immunotherapy, bone-targeted therapy, poly (adenosine diphosphate–ribose) polymerase (PARP) inhibitors, and other novel therapeutic targets (Nuhn et al., 2018).

Programmed cell death 1 (PD-1; CD279) is an inhibitory receptor expressed by tumor-infiltrating lymphocytes (TILs), such as activated T cells, B cells, and natural killer (NK) cells (Pardoll, 2012; Riella et al., 2012). Its ligand, programmed cell death ligand 1 (PD-L1; B7-H1; CD274), is expressed constitutively on specific tumors and immune cells, including T and B cells, dendritic cells (DCs), macrophages, mesenchymal stem cells, and bone marrow-derived mast cells (Riella et al., 2012). PD-1 and PD-L1 are immune check points that limit autoimmunity and the activity of T cells under an inflammatory response to infection (Pardoll, 2012; Wang P. et al., 2017). Anti-PD-1/PD-L1 therapy is a promising immunotherapy that can enhance antitumor immunity and elicit durable clinical responses by blocking the PD-1/PD-L1 signaling pathway (Aghajani et al., 2018). The responses strongly correlated with increased PD-1 expression by TILs and increased PD-L1 expression by tumor cells (Pardoll, 2012). Some published studies reported that PD-L1 expression was a negative predictor for prognosis (Zhang et al., 2016; Aghajani et al., 2018; Keller et al., 2018; Miyama et al., 2018), whereas some other studies manifested inconsistent results (Pardoll, 2012; Wang C. et al., 2017; Huang et al., 2018). Various analyses on diverse tumors have showed that the expression of PD-L1 can associate either with poor prognosis, better prognosis or have no connection with prognosis (Ohigashi et al., 2005; Ghebeh et al., 2006; Wu et al., 2006; Hamanishi et al., 2007; Hino et al., 2010; Pardoll, 2012; Iacovelli et al., 2016; Li et al., 2018). Studies evaluating the prognostic and clinicopathologic significance of PD-L1 expression in PCa are limited, and the curative effect of anti-PD-1/PD-L1 therapy on PCa remains controversial. Therefore, it prompted us to perform a meta-analysis to figure out the prognostic and clinicopathologic significance of PD-L1 in PCa patients, that is to say our meta-analysis aims to find out whether PD-L1 expression of PCa is related to outcome parameters (biochemical recurrence-free survival) and clinicopathologic parameters (e.g., Gleason score). We report this systematic review and meta-analysis following the Preferred Reporting Items for Systematic Reviews and Meta-Analyses (PRISMA) statement (Moher, 2009).

## Methods

### Analysis Workflow

**Figure 1:**
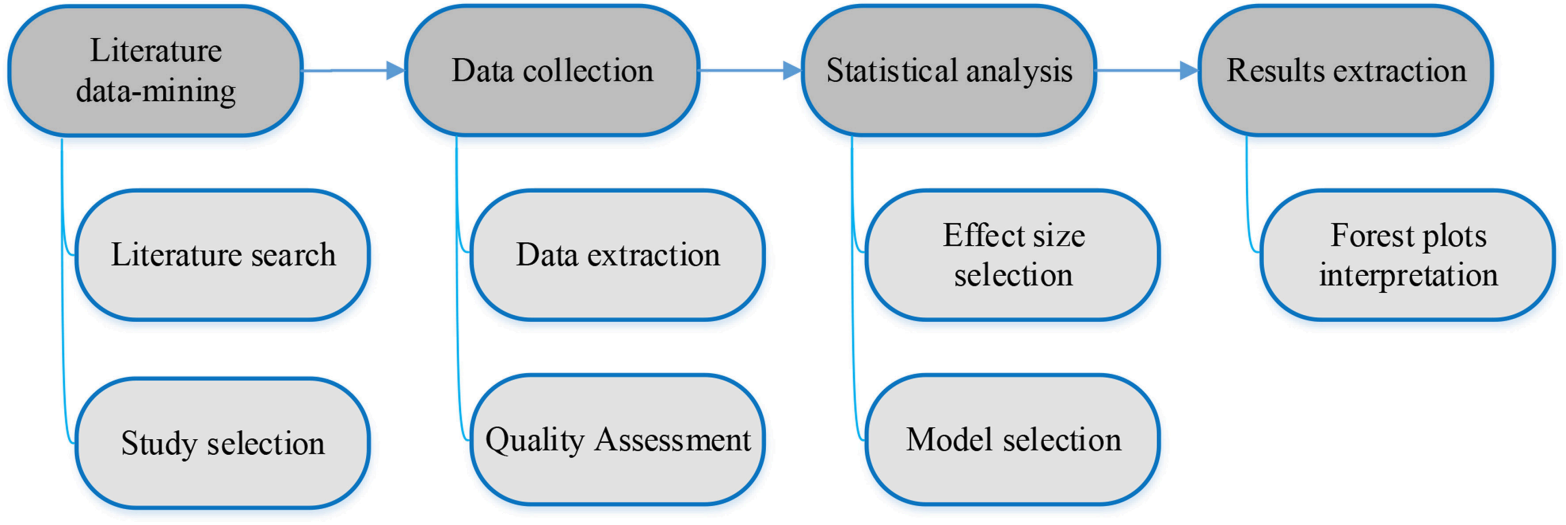
Workflow for meta-analysis of clinicopathologic and prognostic significance of PD-L1 expression in prostate cancer.

Literature data-mining of clinicopathologic and prognostic significance of PD-L1 expression in prostate cancer, data collection, statistical analysis, and associated results extraction followed the workflow depicted in Figure 1 with specifics as provided in the sections below.

### Literature Search

A comprehensive literature search was systematically performed in the PubMed, Cochrane Library, EMBASE, Web of Science, and SCOPUS databases to identify relevant studies up to July 21st, 2018. The following keywords were employed for literature retrieval: (“prostate” or “prostatic”) and (“cancer” or “neoplasm” or “tumor” or “tumor” or “carcinoma”) and (“Programmed Cell Death Ligand 1” or “Programmed Death Ligand 1” or “PD-L1” or “B7-H1” or “CD274” or “Programmed Cell Death 1” or “Programmed Death 1” or “PD-1” or “CD279”). A manual search of potential references was also conducted, and literature in the field of interest was reviewed for additional eligible studies.

### Study Selection

Assessment of every study retrieved was independently examined by two reviewers (Q. Y. Huang and Y. Y. Zhou) for comprehensive evaluation based on the following inclusion criteria: (1) Patients were histologically confirmed as having prostate cancer; (2) PD-L1 protein expression was assessed in prostate cancer tissues; (3) PD-L1 expression was divided into high (positive) and low (negative) categories; (4) studies investigated the association between PD-L1 protein expression and/or mPD-L1 with clinicopathologic features and/or prognosis; (5) studies directly provided hazard ratio (HR) or odd ratio (OR) with corresponding 95% confidence interval (CI), or survival curves/number of patients with specific clinicopathologic features to estimate them; and (6) studies were published in English with available full texts. The exclusion criteria were formulated and improved after we found some studies satisfying our inclusion criteria but could not be included in the final meta-analysis. The exclusion criteria were as follows: (1) studies did not satisfy the inclusion criteria; (2) studies turned out to be reviews, meta-analyses, editorials, case reports, expert opinions, letters, notes, meeting abstracts or proceedings; (3) non-human studies or *in vitro* studies; (4) duplication publications or studies with overlapping data; and (5) studies provided information unable to be pooled. Disagreements about certain studies were resolved by discussion with a third reviewer (YL).

### Data Extraction

The data from the eligible studies were extracted independently by two reviewers (Y. Y. Zhou and Q. Y. Huang) in piloted forms (in duplicate) to tabulate the information, and any disagreements between the two reviewers were resolved with consensus. The following data were collected from each included study: name of the first author, year of publication, country, number of patients, tumor type, technique, PD-L1-positive expression as well as high mPD-L1, cut-off values for PD-L1 positive expression as well as high mPD-L1, the hazard ratios (HRs) and 95% confidence intervals (CIs) for biochemical recurrence-free survival (BCR-FS), and numbers of PD-L1-positive as well as PD-L1-negative patients with (a) age <60 years, (b) age ≥60 years, (c) Gleason score <7, (d) Gleason score ≥7, (e) pathologic stage pT2, (f) pathologic stage pT3-pT4, (g) lymph node metastasis N0, (h) lymph node metastasis N1, (i) preoperative PSA level ≤10 ng/ml, (j) preoperative PSA level >10 ng/ml, (k) androgen receptor-negative (AR-), and (l) androgen receptor-positive (AR+).

### Population, Interventions, Comparators, Outcomes and Study Designs (PICOS)

The population from the study is patients with prostate cancer. PD-L1 expression and/or mPD-L1 was assessed in these patients. PD-L1 status (PD-L1 positive and PD-L1 negative) and mPD-L1 level (high and low) were compared by the endpoint BCR-FS. The correlations of PD-L1 status with age, Gleason score, pathologic stage, lymph node metastasis, preoperative PSA level, and androgen receptor status were evaluated in these patients. The study designs were to evaluate the association between PD-L1 expression/mPD-L1 and prognosis as well as the relationship of PD-L1 expression and age, Gleason score, pathologic stage, lymph node metastasis, preoperative PSA level, and androgen receptor status.

### Quality Assessment

Two investigators (Y. Y. Zhou and Q. Y. Huang) independently conducted the quality assessment of all included studies according to the Newcastle-Ottawa Scale (NOS) criteria to ensure consistency in reviewing and reporting results (Stang, 2010). The NOS consists of the following three parameters of quality: (1) selection: 0–4; (2) comparability: 0–2; and (3) exposure/outcome: 0–3. The maximum of NOS score is nine, with studies scoring greater than five considered to be of high quality. Any discrepancies between reviewers were resolved by consensus.

### Statistical Analysis

Pooled prevalence of PD-L1 in PCa were calculated using Freeman-Tukey double arcsine transformation by R software version 3.5.0. The HR is the ratio of the hazard rates corresponding to the conditions described by two levels of an explanatory variable, and the OR is defined as the ratio of the odds of A in the presence of B and the odds of A without the presence of B, which attempts to quantify the strength of the association between A and B. Pooled HRs with their 95% CIs were implemented to estimate the association between BCR-FS and PD-L1 expression or mPD-L1. Patients were dichotomized by age (<60 years vs. ≥60 years), Gleason score (<7 vs. ≥7), pathologic stage (pT2 vs. pT3-pT4), lymph node metastasis (N0 vs. N1), preoperative PSA level (≤10 ng/mL vs. >10 ng/mL), and androgen receptor status (AR+ vs. AR-) categories of PD-L1 expression by referring to National Comprehensive Cancer Network (NCCN) Guidelines for Prostate Cancer (URL: https://www.nccn.org/professionals/physician_gls/default.aspx#prostate). The dichotomous outcomes were analyzed using the ORs with 95% CI as the summary statistics to evaluate the correlation between PD-L1 expression and the above clinicopathologic parameters. The Review Manager software version 5.3 (Revman, the Cochrane Collaboration; Oxford, England) was used to calculate HR and OR with 95% CIs in this meta-analysis. Heterogeneity is defined as the consequence of methodological and/or statistical diversity among studies and was assessed by the Chi-squared test and I^2^ statistic. I^2^ values less than 25%, from 25 to 50%, and higher than 50% represented low, medium and high heterogeneity, respectively. Statistical tests were all two-sided, with *P-*values < 0.05 considered to be statistically significant. Detailed interpretations of odds ratios, confidence intervals and p-values can be found elsewhere (Tim, 2013).

According to Chapter 13 of the book *Introduction to meta-analysis* (Borenstein et al., 2009), the following three points should be noticed: (a) if the number of studies is very small, then the estimate of the between-studies variance will have poor precision, (b) while the random-effects model is still the appropriate model, we lack the information needed to apply it correctly, and (c) in this case, one option is to perform a fixed-effect analysis. Hence, fixed-effect models were employed for all statistical analyses because the number of our included studies is small.

## Results

### Search Results

**Figure 2:**
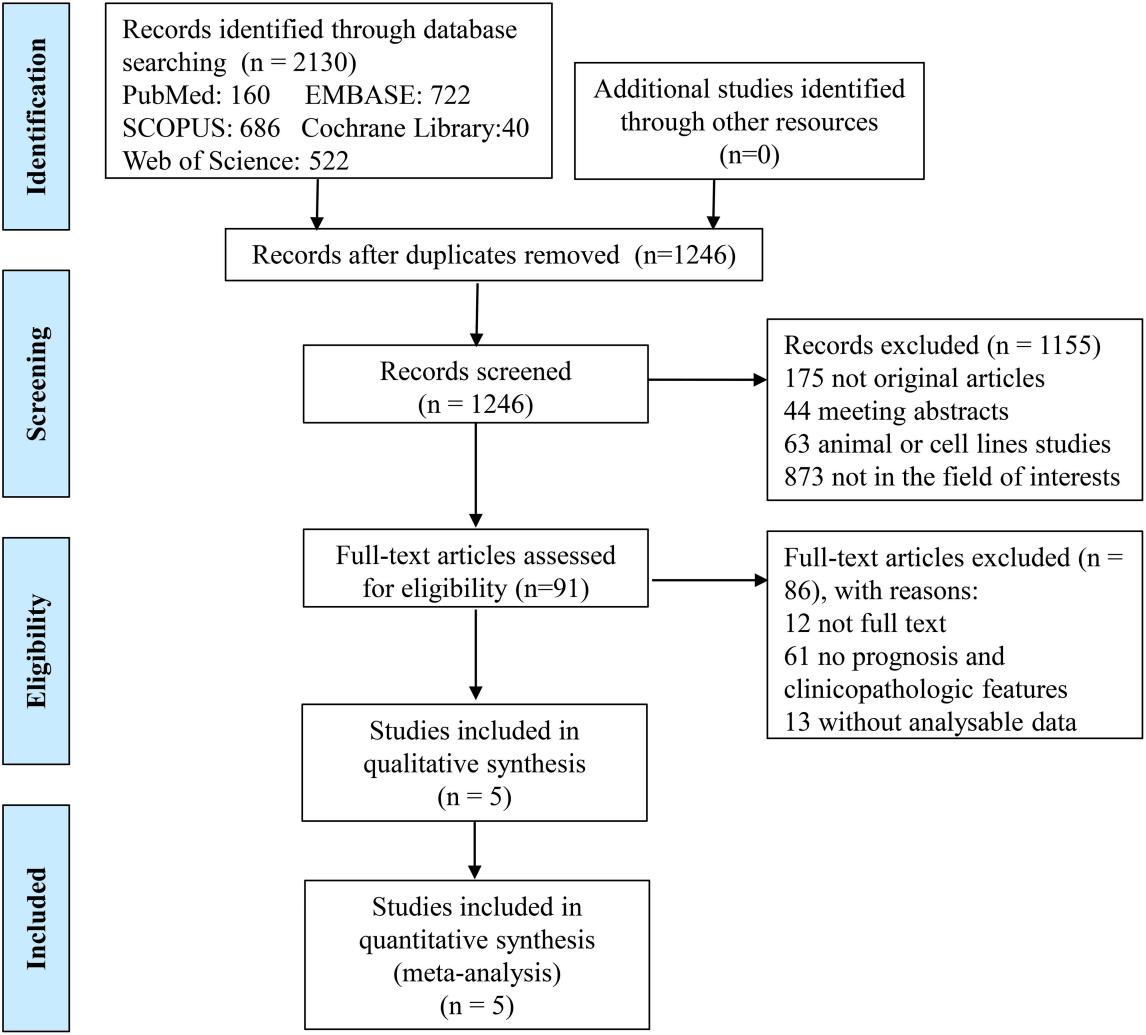
Flow chart of study selection.

In the present study, a total of 2,130 records were identified initially from the five databases, 160 from PubMed, 722 from EMBASE, 686 from SCOPUS, 40 from Cochrane Library, and 522 from Web of Science, by using the search strategy above. After removing the duplicate publications (*n* = 884), the titles and abstracts of all remaining publications (*n* = 1,246) were reviewed, and 1,155 articles were excluded because they were non-original articles (*n* = 175: 131 reviews, 12 meta-analyses, 5 case reports, 14 editorials, 2 letters, 2 expert opinions, 9 notes), meeting abstracts (*n* = 44), animal or cell lines experiments (*n* = 63), or not in the field of interest (*n* = 873). Of 91 remaining studies, 12 full texts were not available and so 79 studies were left. Another 61 studies were excluded for the following reasons: (a) the studies focused on adverse events of anti-PD-1/PD-L1 therapy, the effectiveness of PD-1/PD-L1 inhibitors, the combination therapy with anti-PD-1/PD-L1 therapy plus other treatments, or the influences of other factors on PD-L1 expression; (b) the studies were mechanism studies, pharmacological experiments or ongoing clinical trials; (c) the studies provided no information about outcome parameters (such as overall survival, disease-free survival and progression-free survival) or clinicopathologic features of PD-L1 positive and negative patients. No outcome parameter except biochemical recurrence-free survival (BCR-FS) was found in more than one study, so studies which used the outcome parameters except BCR-FS were excluded. The studies, which provided the clinicopathologic features of PCa patients, but did not provide the respective clinicopathologic features of PD-L1-positive and PD-L1-negative patients, were also excluded. After excluding 13 studies with unanalyzable data mentioned above, five studies were eventually included in the final meta-analysis. A flowchart depicting details of the study selection is shown in Figure 2.

### Study Characteristics

The characteristics of the included studies are summarized in Table 1. The five eligible studies were published between 2009 and 2018: three studies from Germany and two from America. Of note, the article by (Gevensleben et al., 2016a) offered two cohorts: a training cohort and a test cohort, while another article by (Gevensleben et al., 2016b) provided a training cohort and a validation cohort. The validation cohort in 2016 not only evaluated the prognostic value of PD-L1 protein expression, but also the prognostic significance of mPD-L1. Therefore, in total, seven comparisons (from five articles) consisting of 2,272 patients were included in the meta-analysis. Among these articles, PD-L1 expression was detected by using the immunohistochemistry (IHC) staining method in four articles (1,475 cases) and was found in 557 patients (37.8%), with the percentage ranging from 7.7 to 82.4%. As presented in Table 1, different studies adopted different cut-off values to define positive (high) and negative (low) PD-L1 expression. In Ebelt et al. (2009), the estimated number of positively stained cells >50 was considered to be PD-L1-positive. In Calagua et al. (2017) and Haffner et al. (2018), PD-L1 positivity was defined as ≥1% of tumor cells stained positive for PD-L1. In Gevensleben et al. (2016a), PD-L1 expression was dichotomized by median (high = above median, low = below median). In Gevensleben et al. (2016b), PD-L1 DNA methylation dichotomized by an optimized cut-off (mPD-L1_low_ < 0.98% ≤ mPD-L1_high_). The 0.98% here refers to the percentage of DNA methylation. For this pooled analysis, we found PD-L1-positive patients and high mPD-L1 patients according to their own specific cut-off criteria. BCR-FS was implemented as the end point in five comparisons out of two studies (Gevensleben et al., 2016a,b), of which three comparisons were about PD-L1 expression and the other two were comparisons about mPD-L1. Moreover, we compared the prevalence of PD-L1 expression between the following pairs: age <60 years and age ≥60 years (two comparisons), Gleason score <7 and Gleason score ≥7 groups (five comparisons), pathologic stage pT2 and pathologic stage pT3-pT4 groups (five comparisons), lymph node metastasis N0 and N1 (four comparisons), PSA level ≤10 ng/ml and PSA level >10 ng/ml (two comparisons), and androgen receptor-positive and androgen receptor-negative (two comparisons).

**Table 1:** Characteristics of the eligible studies in the meta-analysis.

**References**	**Country**	**No**.	**Tumor**	**Technique**	**Cut-off**	**PD-L1 positive (%)**	**Outcome**	**HR estimation**
Ebelt et al., 2009	Germany	17	Prostate cancer	IHC	Cell counts≥50	14/17 (82.4)	NA	NA
Gevensleben et al., 2016a	Germany	Training cohort: 209	Primary prostate cancer	IHC + TMA	Above median	109/209 (52.2)	BCR-FS	2.37 [1.32–4.25]
Gevensleben et al., 2016a	Germany	Test cohort: 611	Primary prostate cancer	IHC+TMA	Above median	377/611 (61.7)	BCR-FS	1.49 [1.10–2.02]
Gevensleben et al., 2016b	Germany	Validation cohort: 299	Prostate cancer	NA	NA	NA	BCR-FS	2.58 [1.43–4.63]
Gevensleben et al., 2016b	Germany	Validation cohort: 299	Prostate cancer	qPCR	≥0.98%	high mPD-L1: 102/299 (34.1)	BCR-FS	1.90 [1.09–3.31]
Gevensleben et al., 2016b	Germany	Training cohort: 498	Prostate cancer	qPCR	NA	High mPD-L1: 101/498 (20.3)	BCR-FS	2.60 [1.50–4.51]
Calagua et al., 2017	America	130	Prostate cancer	IHC	≥ 1%	18/130(13.8)	NA	NA
Haffner et al., 2018	America	508	Acinar adenocarcinomas of the prostate	IHC	≥ 1%	39/508 (7.7)	NA	NA

*NO, number of patients; NA, not available; IHC, immunohistochemistry; TMA, tissue microarrays; qPCR, quantitative methylation real-time PCR; PD-L1, programmed cell death ligand 1; mPD-L1, PD-L1 DNA methylation; BCR-FS, biochemical recurrence-free survival; HR, hazard ratio*.

Based on the Newcastle-Ottawa quality assessment scale (URL: http://www.ohri.ca/programs/clinical_epidemiology/nosgen.pdf), the NOS scores of the five studies ranged from 6 to 8, with a mean score of 6.8. Thus, these eligible studies were of high quality. The details of the quality assessment are depicted in Tables 2, 3.

**Table 2:** Quality assessment of the case control studies in the meta-analysis.

**Included studies**	**Selection**				**Comparability**	**Exposure**			**Total quality score**
	**S1**	**S2**	**S3**	**S4**	**C**	**E1**	**E2**	**E3**	
Ebelt et al., 2009	a^*^	a^*^	b	b	a^*^	a^*^	a^*^	a^*^	6
Calagua et al., 2017	a^*^	a^*^	a^*^	b	ab^**^	b^*^	a^*^	a^*^	8
Haffner et al., 2018	a^*^	a^*^	c	b	ab^**^	b^*^	a^*^	a^*^	7

*S1, Adequacy of case definition; S2, Representativeness of the cases; S3, Selection of Controls; S4, Definition of Controls; C, Comparability of cases and controls on the basis of the design or analysis; E1, Ascertainment of exposure; E2, Same method of ascertainment for cases and controls; E3, Non-Response rate*.

**Table 3:** Quality assessment of the cohort studies in the meta-analysis.

**Included studies**	**Selection**				**Comparability**	**Outcome**			**Total quality score**
	**S1**	**S2**	**S3**	**S4**	**C**	**O1**	**O2**	**O3**	
Gevensleben et al., 2016a	a^*^	a^*^	c	b	ab^**^	a^*^	a^*^	a^*^	7
Gevensleben et al., 2016b	a^*^	a^*^	c	b	ab^**^	c	a^*^	a^*^	6

*S1, Representativeness of the exposed cohort; S2, Selection of the non-exposed cohort; S3, Ascertainment of exposure; S4, Outcome not present at start of study; C, Comparability of cohorts on the basis of the design or analysis; O1, Assessment of outcome; O2, Length of follow-up; O3, Adequacy of follow-up*.

### Prevalence of PD-L1 Expression in Prostate Cancer

**Figure 3:**
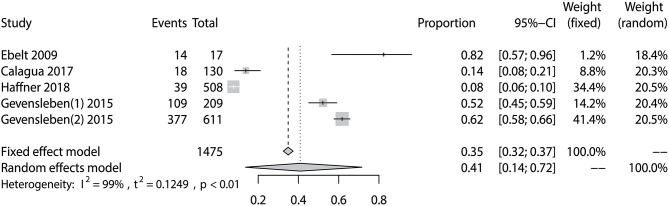
Forest plot showing the pooled prevalence of PD-L1 expression among prostate cancer patients.

The prevalence of PD-L1 expression among prostate cancer patients in the five eligible studies ranged from 7.7 to 82.4% (Table 1). The pooled analysis result gave an overall prevalence of PD-L1 of 35% (fixed effect, 95% CI 0.32 to 0.37) with a significant heterogeneity (*P* < 0.01; *I*^2^ = 99%) (Figure 3).

### PD-L1 and MPD-L1 as Prognostic Factors for Prostate Cancer

**Figure 4:**
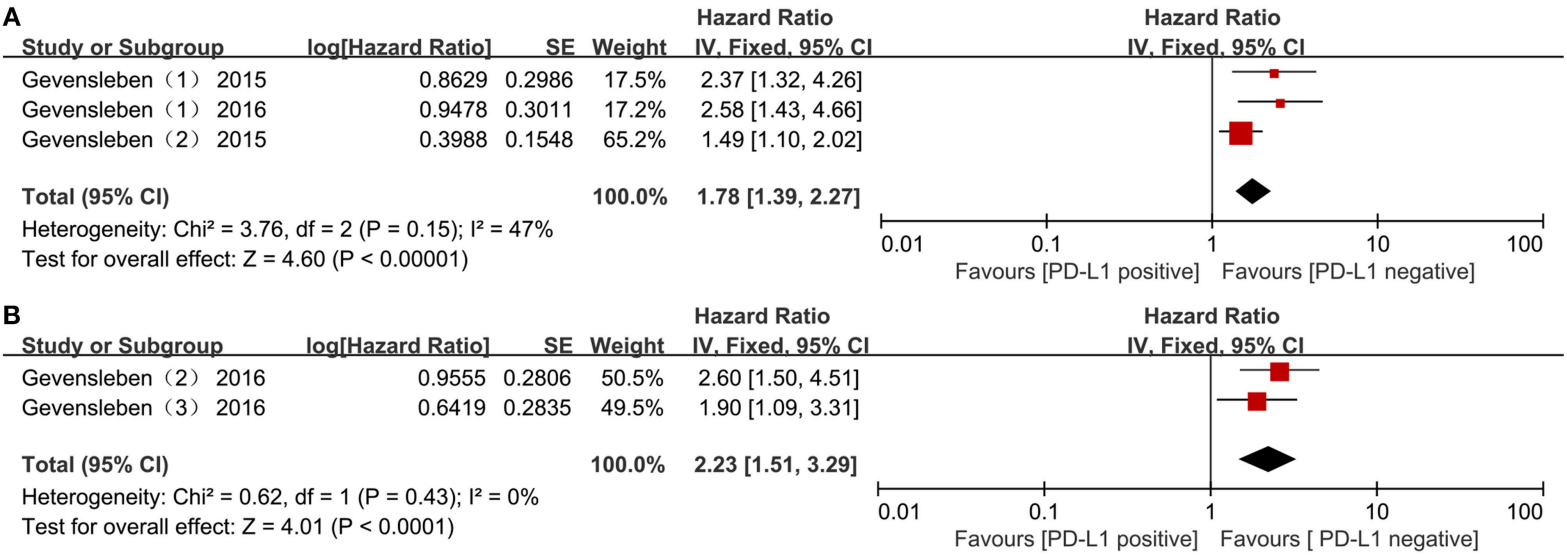
Forest plots evaluating the association between BCR-free survival and PD-L1 protein expression **(A)**, PD-L1 DNA methylation **(B)** in patients with prostate cancer.

Two studies including three comparisons with 1,119 patients reported biochemical recurrence-free survival (BCR-FS). The pooled HR for BCR-FS showed that PD-L1 expression was associated with poor BCR-FS in PCa with statistical significance and a higher level of PD-L1 expression increased the risk of death by 78 % with fixed effects (HR = 1.78; 95 % CI 1.39 to 2.27; *p* < 0.00001) (Figure 4A). There was no significant heterogeneity (Chi^2^ = 3.76, *p* = 0.15; *I*^2^ = 47%).

In addition, an association with statistical significance between high mPD-L1 and the increased risk for BCR was identified (fixed effect, HR = 2.23; 95% CI 1.51 to 3.29; *p* < 0.0001) (Figure 4B), without significant heterogeneity (Chi^2^ = 0.62, *p* = 0.43; *I*^2^ = 0%).

### Correlation Between Pd-L1 Expression and Clinicopathologic Characteristics

#### Age

**Figure 5:**
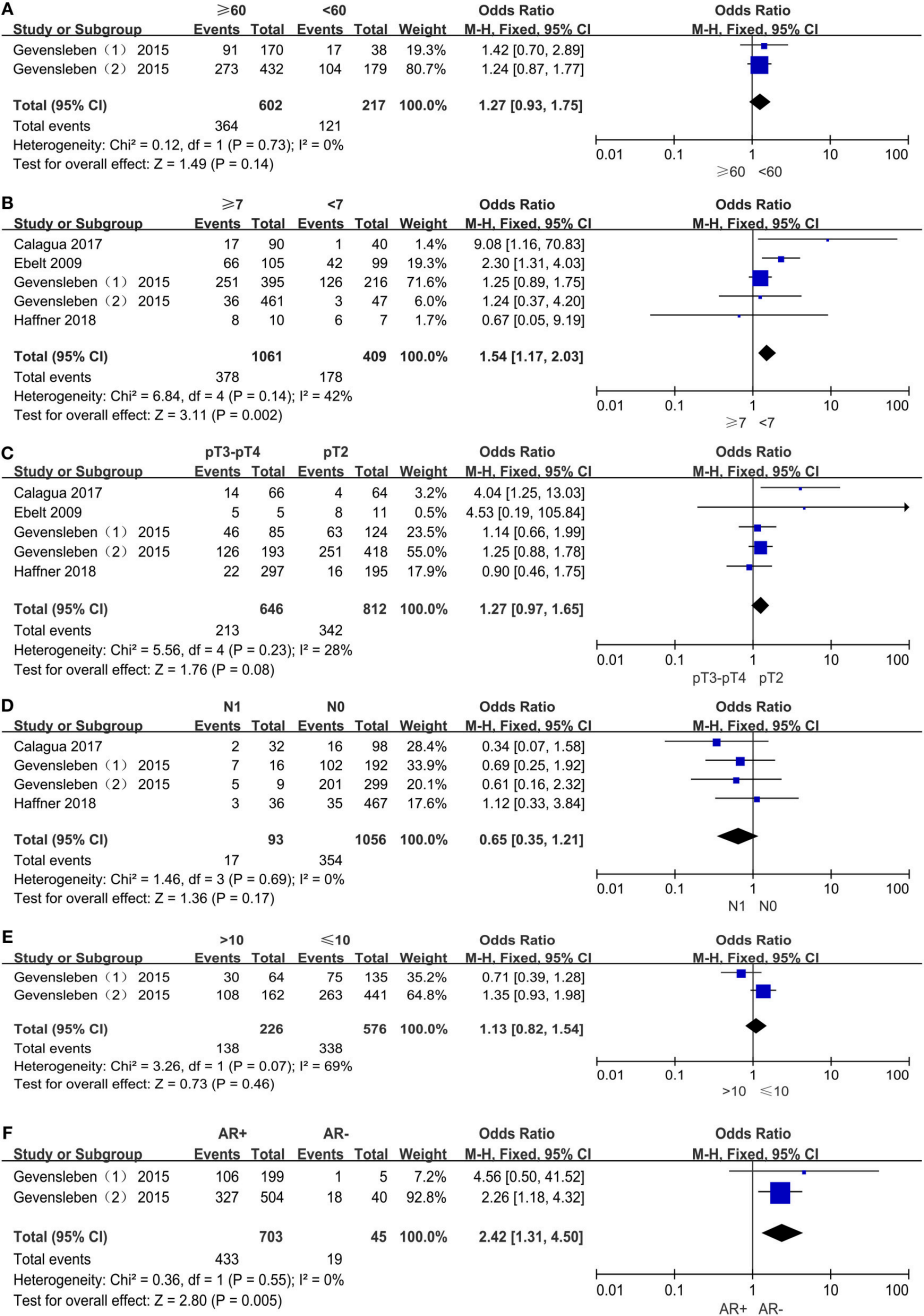
Forest plots for the association between PD-L1 expression and clinicopathologic features: age **(A)**, Gleason score **(B)**, pathologic stage **(C)**, lymph node metastasis **(D)**, preoperative PSA **(E)**, androgen receptor status **(F)**.

We assessed the association between PD-L1 expression and age among 819 patients from two comparisons (Figure 5A). Among 602 older patients (≥60 years), 364 patients (60.5%) were PD-L1 expression positive, and 121 (55.8%) of 217 younger patients (<60 years) were PD-L1 expression positive. Pooled results (*OR* = 1.27; 95% CI 0.93 to 1.75; *P* = 0.14) showed that the odds of positive PD-L1 expression in older patients were 27% higher than in younger patients. However, this result was not statistically significant.

#### Gleason Score

The rate of positive expression of PD-L1 between the groups with Gleason scores ≥7 and <7 was compared in four studies including 1,470 patients (Figure 5B). It was determined that 378 (35.6%) of 1,061 PCa patients with higher Gleason scores and 178 (43.5%) of 409 PCa patients with lower Gleason scores were PD-L1 expression positive, with an odds ratio of 1.54 (95% CI, 1.17 to 2.03; *P* = 0.002). Therefore, the odds of positive PD-L1 expression in PCa patients with higher Gleason scores were 54% higher than those with lower Gleason scores, and this result was statistically significant.

#### Pathologic Stage

A total of 1,458 patients out of four studies were analyzed for the association between PD-L1 expression and pathologic stage (Figure 5C). Then we found that 213 (33.0%) of 646 patients in stage pT3–pT4 and 342 (42.1%) out of 812 patients in stage pT2 were PD-L1 expression positive. The odds of positive PD-L1 expression in patients at stage pT3–pT4 were 27% higher than patients at stage pT2, a result with no statistical significance (*OR* = 1.27, 95% CI 0.97 to 1.65; *P* = 0.08).

#### Lymph Node Metastasis

Three studies comprising 1,149 patients were evaluated for the association between PD-L1 expression and lymph node metastasis (Figure 5D). Of 93 patients with lymph node status N0, 17 (18.3%) were PD-L1 expression positive, and 354 (33.5%) of 1,056 patients with lymph node status N1 were PD-L1 expression positive. The pooled results (*OR* = 0.65, 95% CI 0.35 to 1.21; *P* = 0.17) showed that the odds of positive PD-L1 expression in PCa patients with N0 were 35% lower than those with N1. However, this result was also not statistically significant.

#### Preoperative PSA

Only two comparisons out of one study, which included 802 patients, examined the correlation between PD-L1 expression and preoperative PSA level. Of 226 PCa patients with higher PSA levels (>10 ng/mL), 138 (61.1%) were PD-L1 expression positive and 338 (58.7%) of 576 PCa patients with lower PSA levels (≤10 ng/mL) were PD-L1 expression positive. The odds of positive PD-L1 expression in patients with higher PSA level were 13% higher than those with lower PSA level and this result was not statistically significant (*OR* = 1.13, 95% CI 0.82 to 1.54; *P* = 0.46) (Figure 5E).

#### Androgen Receptor Status

The correlation between PD-L1 expression and androgen receptor status was assessed among two comparisons with 1,200 patients (Figure 5F). Of 703 AR+ patients, 433 (61.6%) were PD-L1-positive, and 19 (42.2%) of 45 AR- patients were PD-L1-positive. The pooled OR (OR = 2.42, 95% CI 1.31 to 4.50; *P* = 0.005) showed a significant association between PD-L1 expression and androgen receptor status. In other words, the odds of positive PD-L1 expression in AR+ patients were 142% higher than AR- patients, with the true population effect between 31 and 350%. This result was statistically significant.

Significant heterogeneity was detected in the analysis of PD-L1 expression with preoperative PSA levels (*P* = 0.07; *I*^2^ = 69%). As for the remaining analyses of PD-L1 expression with age (*P* = 0.73; *I*^2^ = 0%), Gleason score (*P* = 0.14; *I*^2^ = 42%), pathologic stage (*P* = 0.23; *I*^2^ = 28%), lymph node metastasis (*P* = 0.69; *I*^2^ = 0%) and androgen receptor status (*P* = 0.55; *I*^2^ = 0%), there was no evidence of substantial heterogeneity. The number of our included studies is small, hence we performed fixed-effect models for all statistical analyses.

## Discussion

PD-1/PD-L1 antibodies were approved by the US-FDA for multiple tumor types, including melanoma, non-small cell lung cancer, bladder cancer, kidney cancer, etc. (Haffner et al., 2018). However, the therapeutic effect of PD-1/PD-L1 antibodies in prostate cancer remains controversial. The likelihood of antitumor immune response to anti-PD-1 antibody therapy is closely linked to expression of PD-L1 on the tumor cell surface (Brahmer et al., 2010; Pardoll, 2012; Taube et al., 2014). Different tumor types have a wide variety of baseline PD-L1 expression levels (Gatalica et al., 2014; Taube et al., 2014; Haffner et al., 2018). A phase 1 trial (Topalian et al., 2012) assessed the safety and antitumor activity of BMS-936558, a fully human anti-PD-1 monoclonal antibody, in advanced solid tumor patients. Among them, 36% of patients with PD-L1-positive tumors responded to anti-PD-1 antibody, and no objective response was observed in patients with PD-L1-negative tumors, which included PCa patients. Similar results were also found in another phase I study of single-agent anti-PD-1 (MDX-1106) (Brahmer et al., 2010). In our review of several articles, multiple studies had shown that the prevalence of PD-L1 in patients with prostate cancer varied greatly (ranged from 0 to 92%) (Ebelt et al., 2009; Gatalica et al., 2014; Martin et al., 2015; Gevensleben et al., 2016a; Massari et al., 2016; Baas et al., 2017; Calagua et al., 2017; Ness et al., 2017; Haffner et al., 2018; Wang et al., 2018), which may account for the poor efficacy of anti-PD-1/PD-L1 immunotherapy in PCa patients in previous studies. Predictive biomarkers or clinical characteristics are then desperately needed so we can identify patients who will benefit most from anti-PD-1/PD-L1 immunotherapy, and PD-L1 expression has the potential to be a promising predictive biomarker for favorable clinical benefits from therapeutic blockage of PD-1/PD-L1 pathway (Tang and Heng, 2013; Taube et al., 2014).

As far as we know, this present meta-analysis is the first to investigate the clinicopathologic and prognostic significance of PD-L1 expression in prostate cancer. A highly variable frequency of PD-L1 expression has been reported in the included studies measuring the expression of PD-L1 in prostate cancer, which ranged from 7.7 to 82.4% (Ebelt et al., 2009; Gevensleben et al., 2016a; Calagua et al., 2017; Haffner et al., 2018), and the pooled frequency of PD-L1 is 35%. An included study (Gevensleben et al., 2016a) provided the first evidence that the prevalence of PD-L1 expression is very common in primary prostate cancer and is a negative predictor for BCR-free survival. Our pooled results for BCR-FS demonstrated the adverse prognostic value of positive PD-L1 expression and high mPD-L1 in PCa patients. PD-L1 expression could then be considered a risk factor to predict the prognosis of PCa and an effective biomarker to identify the right patient population for anti-PD-1/PD-L1 treatment. There are at least six distinct mechanisms for how PD-L1-expressing cells evade T-cell immunity: inducing (1) apoptosis, (2) anergy or (3) functional exhaustion of T cells, (4) forming a molecular shield to keep lysis off tumor cells, (5) increasing production of the immunosuppressive cytokine IL-10, and (6) facilitating T_Reg_-cell-mediated suppression (Zou and Chen, 2008). These functions of PD-L1 expression might explain its role in cancer immune escape and the relation between tumor progression and poor prognosis. Function-blocking monoclonal antibodies against PD-1 suppress the above reaction and thus activate antitumor immunity.

The fact that both positive PD-L1 expression and high mPD-L1 were significantly connected with undesirable clinical outcomes seems contradictory because DNA methylation is usually perceived to cause gene silencing and thus leads to a decrease of its expression product. A previous study (Gevensleben et al., 2016b) revealed that there was an inverse correlation between mPD-L1 and mRNA transcription but not between mPD-L1 and protein expression in PCa. This finding indicated the research value of post-transcriptional regulatory mechanisms of PD-L1 protein expression. The differential expression of microRNA (miR), the cellular component which can stabilize or degrade mRNA by binding it, plays a significant role in modifying the downstream processing of PD-L1 mRNA, especially miR-197, miR-200, miR-570, miR-34a, and miR-513 (Chen et al., 2015). The intricate correlation between miR, mRNA and mPD-L1 discovered by Gevensleben et al. may therefore explain the interference in the linear translation of PD-L1 mRNA into PD-L1 protein (Gevensleben et al., 2016b). Meanwhile, more advanced research is still needed to unravel the complicated interactions between DNA methylation and PD-L1 expression in PCa.

Recent studies demonstrated that PD-L1 overexpression is related to higher clinical activity in patients with various tumor types receiving anti-PD-1/PD-L1 immunotherapy (Meng et al., 2015). In our analyses, we evaluate the correlation between PD-L1 expression and clinicopathologic features of PCa patients. Based on our pooled results, we provided credible evidence that PCa patients with higher Gleason scores or positive androgen receptor were more likely to have higher levels of PD-L1 expression with statistical significance. These patients are more likely to benefit from blocking the PD-1/PD-L1 pathway. However, the correlations between PD-L1 with age, pathologic stage, lymph node metastasis and preoperative PSA level were not statistically significant.

We performed a Pearson's chi-square test between the positive PD-L1 expression of mCRPC and primary PCa via the data extracted from a previous study evaluating PD-L1 expression in primary and metastatic prostate cancer (Haffner et al., 2018) and found that mCRPC had an increased prevalence of PD-L1 expression compared with primary PCa (*P* < 0.01) (Supplemental Table S1). This result suggests that patients with mCRPC might obtain more favorable clinical benefit from anti-PD-1/PD-L1 immunotherapy rather than patients with primary PCa. Similar statistical analysis was performed based on the data extracted from a study evaluating the effect of neoadjuvant androgen deprivation therapy with abiraterone acetate plus prednisone and leuprolide (Neo-AAPL) on PD-L1 expression in PCa (Calagua et al., 2017), and the difference of the rates of PD-L1 expression between treated and untreated PCa patients was not statistically significant (*p* = 0.062) (Supplemental Table S2) Furthermore, Bishop was the first to put forward that a statistically significantly increase of PD-L1/2^+^ DCs was observed in Enzalutamide-resistant PCa patients compared to those who were naïve (*P* = 0.0037) or those who responded to treatment (*P* = 0.0060) (Bishop et al., 2015). This finding reminds us that patients with Enzalutamide-resistant PCa are more aggressive via suppressing immune responses and more likely to benefit from anti-PD-1/PD-L1 immunotherapy. In addition, a DNA vaccination encoding prostatic acid phosphatase can result in the upregulation of PD-L1 expression on tumor cells of patients with castration-resistant but non-metastatic PCa, hence it provided an in-human rationale for the combination of DNA vaccines with PD-1 blockade for the treatment of PCa patients, which benefits much from vaccines but little from PD-1 antibodies as monotherapies (Rekoske et al., 2016). This combination therapy is currently being examined in patients with mCRPC (NCT02499835).

There are several strengths in this study. First, to our knowledge, this is the first meta-analysis that provides the clinicopathologic and prognostic significance of PD-L1 expression in PCa. Second, our study provides a scientific rationale and direct support for individualized estimations of prognosis for PCa, identification of more aggressive cancer patients, and clinical application of anti-PD-1/PD-L1 immunotherapy. In this way, patients realize precision medicine and individualized treatment. In addition, the study may prompt researchers to design large-cohort clinical trials to further confirm these findings.

We tried our utmost to perform this meta-analysis but there are some limitations of the study that should be acknowledged. First, the quantity of studies included was not big enough to generate more authentic results due to limited published studies. Therefore, more studies are needed to provide more evidence for the prognostic value of PD-L1 and mPD-L1. Second, only articles published in English were included in this meta-analysis. Third, the cut-off values differentiating negative (low) and positive (high) PD-L1 expression varied in different studies. Fourth, the different antibodies used in the included studies might affect the accuracy of the positive rate of PD-L1 expression and might therefore affect the estimation of the prognostic and clinicopathologic value of PD-L1 expression. Previous studies had shown the influence of different antibodies against PD-L1 on the percentage of PD-L1-stained tumor cells (Hirsch et al., 2017; Haffner et al., 2018). Thus, a large multicenter study implementing the same antibody and cut-off value is expected to provide more precise and credible results.

## Conclusion

In conclusion, our meta-analysis confirms the fact that PD-L1 expression and mPD-L1 are significant negative independent prognostic factors in patients with prostate cancer. Moreover, PD-L1 overexpression was statistically significantly linked to high Gleason scores and positive androgen receptor of PCa, while it was also associated with age, pathologic stage, lymph node metastasis and preoperative PSA level but with no statistical significance. This result may guide clinicians in estimating the prognosis of patients individually, identifying patients with poor prognosis, and selecting suitable patients that will obtain favorable clinical benefit to receive anti-PD-1/PD-L1 immunotherapy. This study is expected to attract more practitioners to design retrospective large-cohort studies for the further verification of these findings.

## Author Contributions

YL, QH, YG, and XW: Conception and design; YZ and QH: Collection and assembly of data; YZ and QH: Statistical analysis and interpretation; QH and YL: Manuscript writing; YG and XW: Manuscript revising; All authors: final approval of manuscript.

### Conflict of Interest Statement

The authors declare that the research was conducted in the absence of any commercial or financial relationships that could be construed as a potential conflict of interest.

## Abbreviations

PD-1: programmed cell death 1
PD-L1: programmed cell death ligand 1
mPD-L1: PD-L1 DNA methylation
PCa: prostate cancer
mCRPC: metastatic castration-resistant prostate cancer
NOS: Newcastle Ottawa Quality Assessment Scale
HR: hazard ratio
OR: odds ratio
CI: confidence interval
BCR-FS: biochemical recurrence-free survival
PSA: prostate-specific antigen
IHC: immunohistochemistry
ADT: androgen deprivation therapy
AR: androgen receptor
AR+: androgen receptor-positive
AR-: androgen receptor-negative
PARP: poly (adenosine diphosphate–ribose) polymerase
TILs: tumor-infiltrating lymphocytes
NK cell: natural killer cell
DCs: dendritic cells
miR: microRNA
Neo-AAPL: neoadjuvant androgen deprivation therapy with abiraterone acetate plus prednisone and leuprolide.
