# Supplementary material for: The Clinicopathologic and Prognostic Significance of Programmed Cell Death Ligand 1 (PD-L1) Expression in Patients With Prostate Cancer: A Systematic Review and Meta-Analysis
Source: Front Pharmacol. 2019 Jan 24;9:1494. doi: 10.3389/fphar.2018.01494 (PMC6354218; doi:10.3389/fphar.2018.01494)
Supplement: Supplementary file 1 [file Table_1.docx]

**Supplemental Table S1┃**Chi-Square test of PD-L1 expression in mCRPC and primary PCa.

|  |  |  | risk level | | Total |
| --- | --- | --- | --- | --- | --- |
|  |  |  | Positive | Negative |  |
| Intervention | mCRPC | Count | 39 | 18 | 57 |
|  |  | Expected Count | 50.9 | 6.1 | 57 |
|  |  | % within intervention | 68.4% | 31.6% | 100.0% |
|  | primary PCa | Count | 493 | 46 | 539 |
|  |  | Expected Count | 481.1 | 57.9 | 539.0 |
|  |  | % within intervention | 91.5% | 8.5% | 100.0% |
| Total |  | Count | 532 | 64 | 596 |
|  |  | Expected Count | 532.0 | 64.0 | 596.0 |
|  |  | % within intervention | 89.3% | 10.7% | 100.0% |
| Pearson Chi-Square test | Value | 28.560^a^ |  |  |  |
|  | Asymp. Sig. (2-sided) | 0.000 |  |  |  |
