# Supplementary material for: The Clinicopathologic and Prognostic Significance of Programmed Cell Death Ligand 1 (PD-L1) Expression in Patients With Prostate Cancer: A Systematic Review and Meta-Analysis
Source: Front Pharmacol. 2019 Jan 24;9:1494. doi: 10.3389/fphar.2018.01494 (PMC6354218; doi:10.3389/fphar.2018.01494)
Supplement: Supplementary file 2 [file Table_2.docx]

**Supplemental Table S2┃**Chi-Square test of PD-L1 expression in Neo-AAPL Treated patients and matched untreated patients.

|  |  |  | risk level | | Total |
| --- | --- | --- | --- | --- | --- |
|  |  |  | Positive | Negative |  |
| Intervention | Neo-AAPL Treated | Count | 41 | 3 | 44 |
|  |  | Expected Count | 38.0 | 6.0 | 44.0 |
|  |  | % within intervention | 93.2% | 6.8% | 100.0% |
|  | matched untreated | Count | 35 | 9 | 44 |
|  |  | Expected Count | 38.0 | 6.0 | 44.0 |
|  |  | % within intervention | 79.5% | 20.5% | 100.0% |
| Total |  | Count | 76 | 12 | 88 |
|  |  | Expected Count | 76.0 | 12.0 | 88.0 |
|  |  | % within intervention | 86.4% | 13.6% | 100.0% |
| Pearson Chi-Square test | Value | 3.474 |  |  |  |
|  | Asymp. Sig. (2-sided) | 0.062 |  |  |  |
